# Symmetry selective directionality in near-field acoustics

**DOI:** 10.1093/nsr/nwaa040

**Published:** 2020-03-14

**Authors:** Yang Long, Hao Ge, Danmei Zhang, Xiangyuan Xu, Jie Ren, Ming-Hui Lu, Ming Bao, Hong Chen, Yan-Feng Chen

**Affiliations:** Center for Phononics and Thermal Energy Science, China-EU Joint Center for Nanophononics, Shanghai Key Laboratory of Special Artificial Microstructure Materials and Technology, School of Physics Sciences and Engineering, Tongji University, Shanghai 200092, China; Department of Materials Science and Engineering, College of Engineering and Applied Sciences and National Laboratory of Solid State Microstructures, Nanjing University, Nanjing 210093, China; Center for Phononics and Thermal Energy Science, China-EU Joint Center for Nanophononics, Shanghai Key Laboratory of Special Artificial Microstructure Materials and Technology, School of Physics Sciences and Engineering, Tongji University, Shanghai 200092, China; Department of Materials Science and Engineering, College of Engineering and Applied Sciences and National Laboratory of Solid State Microstructures, Nanjing University, Nanjing 210093, China; Key Laboratory of Noise and Vibration Research, Institute of Acoustics, Chinese Academy of Sciences, Beijing 100190, China; Center for Phononics and Thermal Energy Science, China-EU Joint Center for Nanophononics, Shanghai Key Laboratory of Special Artificial Microstructure Materials and Technology, School of Physics Sciences and Engineering, Tongji University, Shanghai 200092, China; Department of Materials Science and Engineering, College of Engineering and Applied Sciences and National Laboratory of Solid State Microstructures, Nanjing University, Nanjing 210093, China; Collaborative Innovation Center of Advanced Microstructures, Nanjing University, Nanjing 210093, China; Key Laboratory of Noise and Vibration Research, Institute of Acoustics, Chinese Academy of Sciences, Beijing 100190, China; Center for Phononics and Thermal Energy Science, China-EU Joint Center for Nanophononics, Shanghai Key Laboratory of Special Artificial Microstructure Materials and Technology, School of Physics Sciences and Engineering, Tongji University, Shanghai 200092, China; Department of Materials Science and Engineering, College of Engineering and Applied Sciences and National Laboratory of Solid State Microstructures, Nanjing University, Nanjing 210093, China; Collaborative Innovation Center of Advanced Microstructures, Nanjing University, Nanjing 210093, China

**Keywords:** acoustics, near field, symmetry

## Abstract

Understanding unidirectional and topological wave phenomena requires the unveiling of intrinsic geometry and symmetry for wave dynamics. This is essential yet challenging for the flexible control of near-field evanescent waves, highly desirable in broad practical scenarios ranging from information communication to energy radiation. However, exploitations of near-field waves are limited by a lack of fundamental understanding about inherent near-field symmetry and directional coupling at sub-wavelengths, especially for longitudinal waves. Here, based on the acoustic wave platform, we show the efficient selective couplings enabled by near-field symmetry properties. Based on the inherent symmetry properties of three geometrically orthogonal vectors in near-field acoustics, we successfully realize acoustic Janus, Huygens, spin sources and quadrupole hybrid sources, respectively. Moreover, we experimentally demonstrate fertile symmetry selective directionality of those evanescent modes, supported by two opposite meta-surfaces. The symmetry properties of the near-field acoustic spin angular momenta are revealed by directly measuring local vectorial fields. Our findings advance the understanding of symmetries in near-field physics, supply feasible approaches for directional couplings, and pave the way for promising acoustic devices in the future.

## Teaser

It is essential yet challenging to achieve flexible control of near-field evanescent waves, highly desirable in broad practical scenarios. Here, we show the efficient symmetry selective couplings for near-field acoustics

## INTRODUCTION

The near-field waves are confined in the close region around objects, that is scatters, sources and waveguides, which usually decay exponentially with distance from objects [1]. Owing to their highly spatial confinement and intensive energy density, the near-field waves have been widely exploited in efficient communication [2,3] and wireless energy transfer [4–6]. Among these practical applications, one of the main goals is to achieve the selective directional coupling [7]. However, the near-field based selective coupling is often difficult to realize because it does not have directional properties like the far-field propagating wave. For example, one can easily select the far-field wave receiver by pointing the source to it directly or exploiting the meta-surface to introduce extra phase gradients to the propagation phase [8–11]. In contrast, the selective directional couplings of near fields have so far been highly based on the chiral incident waves [12] and the resonant anisotropic structures [13–17], which are restricted to optical wave systems of divergence-free transverse nature. Several selective-coupling proposals about unidirectional particle scatterings on planar interfaces have been also discussed [1,18,19], which just reflect the functions of spin-orbit coupling [20,21] and quantum spin Hall effect [22] for optical surface evanescent modes. Near-field geometrical relations are also drawing much attention recently [23] and some transverse electromagnetic excitation sources of selective directionality are proposed in optics [24], which are highly dependent on the electric and magnetic component matching.

Along with the transverse wave, the curl-free longitudinal wave as the other fundamental component in wave families also plays an indispensable role in many engineering applications or practical scenarios, i.e. acoustic wave, compression elastic wave and P-type seismic wave. Yet, the exploitations of near-field longitudinal waves are severely limited by a lack of fundamental understanding about symmetry essences. And some geometrical and dynamic properties about longitudinal waves are usually overlooked, for example the acoustic spin angular momentum, which was not considered until recently [25–27]. Therefore, despite many optical near-field research works [23,24,28], mapping the near-field research from electromagnetics to acoustics is not a transparent and trivial task.

It is worth emphasizing the many fundamental differences between acoustics and optics: (1) A longitudinal acoustic wave has different physical origin, dynamic equation and geometrical properties from a transverse optical wave. The acoustic wave originates from the regular collective motions of molecules, satisfies the dynamic equation based on Newtonian mechanics, and has a geometrical curl-free nature. In contrast, the optical wave is described by relativistic Maxwell equations, composed of the synchronized oscillated electric and magnetic fields, of a geometrical divergence-free nature. (2) Basic properties of source elements are totally different. There is a monopole for acoustics, but, generally, this does not hold for optics. Besides, unlike acoustics, there are two dual types of multipoles in optics: the electric and magnetic multipoles. (3) There is NO optical magnetic vector field counterpart in acoustics. As such, some necessary requirements of electric and magnetic fields in optical sources [24,28] cannot be simply mapped into acoustics. Thus, the acoustical near-field sources must be achieved without the help of magnetic fields. (4) The idea for near-field acoustic sources will reversely provide more insights on pure electric (magnetic-free) near-field optical sources, to surmount the difficulty of manipulating magnetic fields because of the usually weak magnetic responses of optical media. All of these differences reflect the fact that the near-field research in longitudinal acoustic waves is a meaningful research field to explore, not a simple extension of related optical works, and will even provide new insight for other near-field waves.

In this paper, based on the acoustic wave platform, we unveil the inherent symmetry and geometric properties of near-field waves experimentally. Based on symmetry analysis, it is possible to achieve selective near-field longitudinal wave coupling. The near-field acoustics can be universally associated with three geometrically orthogonal physical vectors: time-average energy flow (Poynting vector), reactive power and spin angular momentum (SAM) [25–27], even for longitudinal waves of a curl-free nature. Based on their symmetry properties and geometrical orthogonal conditions, we realize highly selective near-field couplings for the acoustic ‘plasmonics’: Janus source, Huygens source, and spin source. By exploiting two opposite comb-like meta-surfaces that support four different evanescent modes, we experimentally demonstrate the rich near-field symmetries by selectively exciting different mode branch pairs. Moreover, the SAM densities of the near-field acoustics have been fully discussed and verified experimentally in terms of odd/even symmetry properties. Based on verified symmetry discussions, a quadrupole hybrid source is proposed, which can achieve one-side uni-directional excitations out of four branches. Our work provides a new opportunity to achieve symmetry selective directionality of near-field modes, regardless of the transverse or longitudinal wave systems.

## RESULTS

**Figure 1.**
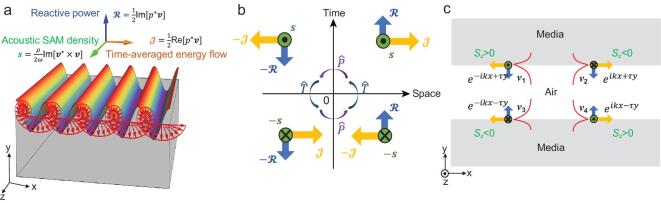
Geometry and symmetry properties of near-field waves. (a) The evanescent wave can be supported on the interface between conducting (air in the acoustic case) and insulating media (gray block). The pressure field of surface wave is plotted as the rainbow color wave and its velocity field is represented as the red arrow. There are three vector physical quantities describing the near-field properties: time-average energy flow $\boldsymbol {\mathcal {J}}$, reactive power $\boldsymbol {\mathcal {R}}$ and acoustic SAM density $\boldsymbol {s}$. (b) The three orthogonal vector quantities : $\boldsymbol {\mathcal {J}}$ (yellow), $\boldsymbol {\mathcal {R}}$ (blue), and $\boldsymbol {s}$ (green) have different symmetries under the $\hat{P}\hat{T}$, $\hat{T}$, $\hat{P}$ transformation. $\boldsymbol {\mathcal {J}}$ is $\hat{P}$-antisymmetric, $\hat{T}$-antisymmetric, yet $\hat{P}\hat{T}$-symmetric; $\boldsymbol {\mathcal {R}}$ is $\hat{P}$-antisymmetric, yet $\hat{T}$-symmetric; $\boldsymbol {s}$ is $\hat{P}$-symmetric, yet $\hat{T}$-antisymmetric. The space mirror operation $\boldsymbol {r} \rightarrow -\boldsymbol {r}$ and $-\boldsymbol {r} \rightarrow \boldsymbol {r}$ will correspond to $\hat{P}$ operation, the time mirror operation *t* → −*t* and −*t* → *t* will correspond to $\hat{T}$ operation. (c) Four evanescent wave modes on the interface have been exploited to demonstrate the symmetry properties in the near fields. The selective couplings between the acoustic source and these near field modes reflect the intrinsic symmetric characteristics and geometrical orthogonality. We can see that $\hat{P} \boldsymbol {v}_4 \hat{P}^{-1} = \boldsymbol {v}_1$, $\hat{T} \boldsymbol {v}_4 \hat{T}^{-1} = \boldsymbol {v}_3$, $\hat{P}\hat{T} \boldsymbol {v}_4 (\hat{P}\hat{T})^{-1} = \boldsymbol {v}_2$ where $\boldsymbol {v}_i$ is the acoustic field profile for the *i*-th branch. Clearly, the mode pair $\boldsymbol {v}_4+ \boldsymbol {v}_1$ has the $\hat{P}$-symmetry with invariant SAM $\boldsymbol {s}$, the mode pair $\boldsymbol {v}_4+ \boldsymbol {v}_3$ has the $\hat{T}$-symmetry with invariant reactive power $\boldsymbol {\mathcal {R}}$, and the mode pair $\boldsymbol {v}_4+ \boldsymbol {v}_2$ has the $\hat{P}\hat{T}$-symmetry with invariant Poynting vector $\boldsymbol {\mathcal {J}}$. It is worth noting that although exemplified in acoustics of longitudinal nature, the geometry and symmetry discussed here are universal for general waves.

Considering the common harmonic near-field acoustic wave form, $\boldsymbol {v} \propto e^{i k x - \tau y} e^{-i\omega t}$ and its longitudinal nature $\nabla \times \boldsymbol {v}=0$, one can obtain the polarized velocity state of near-field acoustics as $\boldsymbol {v} = v_0 (1, i \frac{\tau }{k}, 0) e^{i k x - \tau y}$, where $v$_0_ is the amplitude of acoustic velocity field, τ is the decay rate of the evanescent wave in *y* direction, and ω is the frequency. To describe the dynamic features of near-fields, we can exploit three vectorial quantities [23,24]: time-averaged energy flow (Poynting vector) $\boldsymbol {\mathcal {J}} = \frac{1}{2} {\rm Re}[p^*\boldsymbol {v}]$, reactive power $\boldsymbol {\mathcal {R}} = \frac{1}{2} {\rm Im}[p^*\boldsymbol {v}]$ and acoustic SAM density [25–27] $\boldsymbol {s} = \frac{\rho _0}{2\omega } {\rm Im}[\boldsymbol {v}^*\times \boldsymbol {v}]$ (see Supplementary data), shown in Fig. 1(a), where ρ_0_ is the mass density and *p* is the pressure field. Considering their parity ($\hat{P}: \boldsymbol {r}\rightarrow -\boldsymbol {r}$), time-reversal ($\hat{T}: t\rightarrow -t$) and parity-time ($\hat{P}\hat{T}: \boldsymbol {r}\rightarrow -\boldsymbol {r}, t\rightarrow -t$) transformations, respectively, one can obtain that:

(1) \begin{eqnarray*} \hat{P}\boldsymbol {\mathcal {J}}\hat{P}^{-1} = -\boldsymbol {\mathcal {J}}, \hat{P}\boldsymbol {\mathcal {R}}\hat{P}^{-1} = - \boldsymbol {\mathcal {R}}, \hat{P}\boldsymbol {s}\hat{P}^{-1} = \boldsymbol {s} \nonumber\\ \hat{T}\boldsymbol {\mathcal {J}}\hat{T}^{-1} = -\boldsymbol {\mathcal {J}}, \hat{T}\boldsymbol {\mathcal {R}}\hat{T}^{-1} = \boldsymbol {\mathcal {R}}, \hat{T}\boldsymbol {s}\hat{T}^{-1} = -\boldsymbol {s} \nonumber\\ \hat{P}\hat{T}\boldsymbol {\mathcal {J}}(\hat{P}\hat{T})^{-1} = \boldsymbol {\mathcal {J}}, \hat{P}\hat{T}\boldsymbol {\mathcal {R}}(\hat{P}\hat{T})^{-1} = -\boldsymbol {\mathcal {R}} \nonumber\\ \hat{P}\hat{T}\boldsymbol {s}(\hat{P}\hat{T})^{-1} = -\boldsymbol {s}. \end{eqnarray*}

We can see that these quantities have different symmetry properties from their inherent geometrical properties, as shown in Fig. 1(b). Clearly, the Poynting vector $\boldsymbol {\mathcal {J}}$, the reactive power $\boldsymbol {\mathcal {R}}$, and the SAM $\boldsymbol {s}$, are individually invariant under parity-time ($\hat{P}\hat{T}$), time-reversal ($\hat{T}$) and parity ($\hat{P}$) transformations, respectively. Owing to the inherent geometry and symmetry of these vectorial quantities, the near-field acoustic modes carry their symmetry properties as well, as shown in Fig. 1(c), where the waveguide evanescent waves are taken as examples. Based on the same symmetry analysis, we can obtain that $\hat{P} \boldsymbol {v}_4 \hat{P}^{-1} = \boldsymbol {v}_1$, $\hat{T} \boldsymbol {v}_4 \hat{T}^{-1} = \boldsymbol {v}_3$, $\hat{P}\hat{T} \boldsymbol {v}_4 (\hat{P}\hat{T})^{-1} = \boldsymbol {v}_2$ where $\boldsymbol {v}_i$ is the acoustic field profile for the *i*-th branch. From these symmetries, we can see that the mode pair $\boldsymbol {v}_4+ \boldsymbol {v}_1$ has the $\hat{P}$-symmetry with invariant SAM $\boldsymbol {s}$, $\hat{P}(\boldsymbol {v}_1+\boldsymbol {v}_4)\hat{P}^{-1} = \boldsymbol {v}_1+\boldsymbol {v}_4$; the mode pair $\boldsymbol {v}_4+ \boldsymbol {v}_3$ has the $\hat{T}$-symmetry with invariant reactive power $\boldsymbol {\mathcal {R}}$, $\hat{T}(\boldsymbol {v}_3+\boldsymbol {v}_4)\hat{T}^{-1} = \boldsymbol {v}_3+\boldsymbol {v}_4$; the mode pair $\boldsymbol {v}_4+ \boldsymbol {v}_2$ has the $\hat{P}\hat{T}$-symmetry with invariant Poynting vector $\boldsymbol {\mathcal {J}}$, $\hat{P}\hat{T}(\boldsymbol {v}_2+\boldsymbol {v}_4)(\hat{P}\hat{T})^{-1} = \boldsymbol {v}_2+\boldsymbol {v}_4$. One of the key points of our work is to achieve selective directional couplings of the evanescent modes based on their vector geometry and symmetry relations.

According to the acoustic radiation theory [29], acoustic sources may be regarded as the superposition of acoustic monopole, dipole and quadrupole, represented in a general form as:

(2) \begin{equation*} \boldsymbol {Q}_s = \alpha M + \boldsymbol {\beta }\cdot \boldsymbol {D}+ \overline{\overline{\gamma }}:\overline{\overline{T}}, \end{equation*}

where $\boldsymbol {Q}_s$ represents the wave vector of acoustic source; *M*, $\boldsymbol {D}$ and $\overline{\overline{T}}$ denote the acoustic monopole, dipole and quadrupole; α, $\boldsymbol {\beta }$ and $\overline{\overline{\gamma }}$ are the corresponding constant, vector and matrix coefficients, respectively. Acoustic monopole, dipole and quadrupole are well-known sources that are usually exploited to excite fertile acoustic far-field wave modes [29]. For example, monopole excites far-fields equally for all directions, dipole will not radiate equally and make sound cancel in some regions, and quadrupole has a clover-leaf like radiation pattern. These complex far-field radiation behaviours can be understood well by their inner geometry and symmetry properties, yet the near-field interference behaviors of their super-positions have little been discussed.

Concretely, we introduce three near-field sources based on the geometry and symmetry properties of the near-field acoustics: (i) *Acoustic Janus source*:

(3) \begin{equation*} \boldsymbol {Q}_{\text{Janus}} = M \pm D_y, \end{equation*}

which can be constructed by in-phase combination of acoustic monopole and dipole. This composite source can radiate symmetric far-field waves, but will selectively couple with only single-side near-field modes leaving the opposite side uncoupled. This near-field Janus source is associated with the reactive power $\boldsymbol {\mathcal {R}}$, whose $\hat{P}$-antisymmetry and $\hat{T}$-symmetry properties are the physical reasons for ‘two face’ properties of acoustic Janus source similar to the optical version [24]. (ii) *Acoustic Huygens source*:

(4) \begin{equation*} \boldsymbol {Q}_{\text{Huygens}}= M \pm i D_x, \end{equation*}

which is the π/2 out of phase combination of acoustic monopole and dipole. It can be associated with the time-averaged Poynting energy flow $\boldsymbol {\mathcal {J}}$ of $\hat{P}\hat{T}$-symmetry and induce the far-field wave in a given direction (more details in Supplementary data), which is still valid for near-field directional couplings. (iii) *Acoustic spin source*:

(5) \begin{equation*} \boldsymbol {Q}_{\text{spin}} = D_x \pm i D_y, \end{equation*}

which can be associated with non-zero SAM density $\boldsymbol {s} \ne 0$ that is of $\hat{T}$-antisymmetry and $\hat{P}$-symmetry properties. The transverse SAM of the near field acoustics is induced by their circularly polarized velocity fields, only recently revealed for longitudinal waves [25–27].

**Figure 2.**
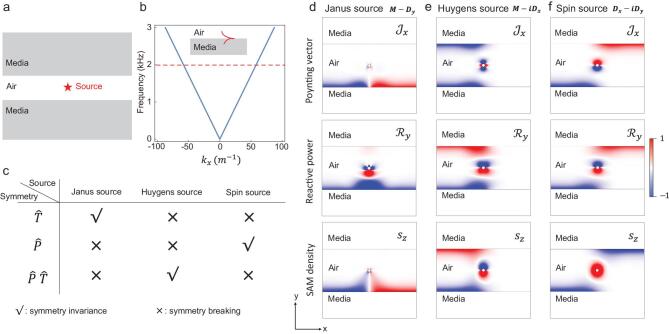
Symmetry properties by Janus, Huygens and spin sources. (a) The source is placed on the center between two effective media: ρ_eff_ = −1.5 kg/m^2^, *c*_eff_ = 500 m/s. The gap between two media is 10 cm. (b) The dispersion of surface evanescent waves. The excitation frequency is *f* = 2 kHz. (c) The symmetry properties of these three acoustic sources. $\hat{P}M\hat{P}^{-1}=M$, $\hat{T}M\hat{T}^{-1}=M$, $\hat{P}\boldsymbol {D}\hat{P}^{-1}=-\boldsymbol {D}$, $\hat{T}\boldsymbol {D}\hat{T}^{-1}=\boldsymbol {D}$, $\hat{T}i\hat{T}^{-1}=-i$. $\hat{T}\boldsymbol {Q}_{\rm Janus}\hat{T}^{-1} = \boldsymbol {Q}_{\rm Janus}$, $\hat{P}\hat{T}\boldsymbol {Q}_{\rm Huygens}(\hat{P}\hat{T})^{-1} = \boldsymbol {Q}_{\rm Huygens}$, $\hat{P}\boldsymbol {Q}_{\rm Spin}\hat{P}^{-1} = e^{i\pi }\boldsymbol {Q}_{\rm Spin}$. It should be mentioned that the extra phase θ ∈ [0, 2π] for the sources will not change their excitation behaviours, namely $e^{i\theta }\boldsymbol {Q}_s$ will be the same as $\boldsymbol {Q}_s$. The physical quantities $\mathcal {J}_x$, $\mathcal {R}_y$ and *s*_$z$_ have been plotted for these sources: Janus source (d), Huygens source (e), spin source (f). We plot these value distributions in the air region.

Based on the near-field modes depicted in Fig. 1, we calculate the pressure field of excited acoustic evanescent waves to show the symmetry-selected directionality in Fig. 2. We place above three sources into the central air sandwiched between two media, as shown in Fig. 2(a). The medium here is an acoustic meta-material with effective negative mass density and effective phase velocity, which can support surface evanescent wave modes on the interface with air [30] [see Fig. 2(b)]. The symmetry properties of the three sources are summarized in Fig. 2(c). We have calculated the $\boldsymbol {\mathcal {J}},\boldsymbol {\mathcal {R}}$ and $\boldsymbol {s}$ for these three sources, as shown in Fig. 2(d-f). From the results, we can see that Janus, Huygens and spin sources will excite the modes with the same $\boldsymbol {\mathcal {R}}$, $\boldsymbol {\mathcal {J}}$ and $\boldsymbol {s}$, respectively.

Besides the symmetry-based analysis, the directional properties can also be described by calculating the coupling coefficient *C* for surface wave modes, following the similar spirit of Fermi golden rule. We introduce the description vector of the acoustic wave field $\mathcal {F} = (v_x,v_y,v_z,-\frac{i}{\rho _0 v_0}p)$ and the source vector $\mathcal {E} = (\beta _x,\beta _y,\beta _z,\alpha )$. According to the Fermi golden rule, the coupling coefficient *C* can be described as: $C \sim \mathcal {F}^* \cdot \mathcal {E}$. For the Janus source, its coupling strength is |*C*|^2^ ∼ (*k*_0_ ∓ τ)^2^ with *k*_0_ = ω/*c*_0_, which is strongly associated with the decay rate τ of the evanescent wave from the $\hat{T}$-symmetry of $\boldsymbol {\mathcal {R}}$. For the Huygens source, |*C*|^2^ ∼ (*k*_0_ ± *k*)^2^, which reflects the relation between the coupling and energy flow from the $\hat{P}\hat{T}$-symmetry of $\boldsymbol {\mathcal {J}}$. For the spin source, $|C|^2 \sim (1 \pm \frac{\tau }{k})^2 \propto (1 \pm \frac{1}{k^2}\boldsymbol {s}\cdot \boldsymbol {e}_z)^2$ is associated with the acoustic SAM density $\boldsymbol {s}$, which reflects the spin-momentum locking and the $\hat{P}$-symmetry of $\boldsymbol {s}$ (see Method ‘The coupling theory of acoustic sources’, and ‘Acoustic spin angular momentum’).

**Figure 3.**
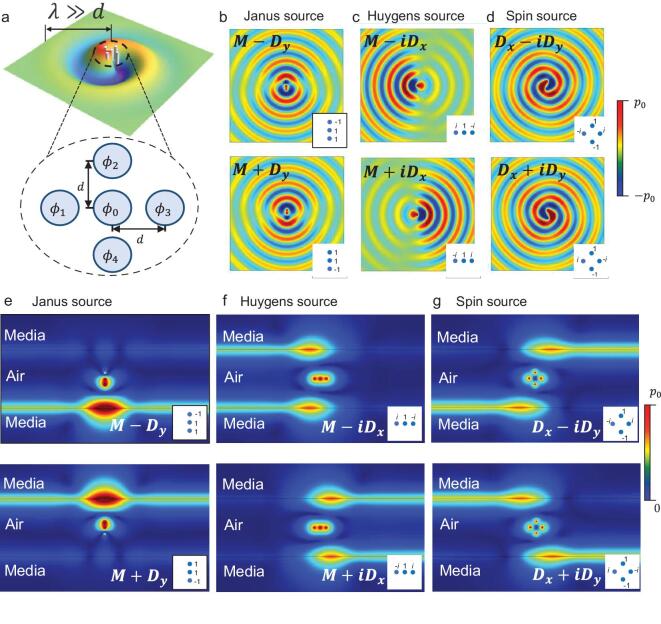
Experimental proposal of Janus, Huygens and spin sources. (a) Five acoustic speakers (monopole) can be exploited to achieve these acoustic sources. The insets in plots (b-g) describe the corresponding strength and phase of these speakers. The radiative far-field behaviours: (b) Janus source, (c) Huygens source, (d) spin source. The pressure fields have been plotted. The near-field excitation behaviours (same materials in Fig. 2): (e) Janus source, (f) Huygens source, (g) spin source. The pressure field amplitudes have been plotted. Here, *d* = 1 cm, and the frequency *f* = 2 kHz.

To realize these sources in experiments, we exploit five speakers placed in a cross bending shape and excite them with specific amplitude and phase settings {φ_*i*_}, as shown in Fig. 3(a), which will be functional to achieve arbitrary combinations of acoustic monopole and dipole at sub-wavelength scale (see Method ‘Experimental proposal for arbitrary acoustic sources’). The amplitude of effective monopole and dipoles are related to the concrete geometrical settings of the speaker array. The excitation schemes for these monopoles to induce the target sources are shown in the inset of figures in Fig. 3. For example, *i* means the normalized amplitude with π/2 phase delay, −1 means the normalized amplitude with π phase delay and the speakers that are not used can be regarded as the zero amplitude (not shown in the insets). The far-field radiation modes of these sources are shown in Fig. 3(b-d). In Fig. 3(e), we can see that the Janus source is achieved to selectively couple to a single side surface mode. The phenomenon originates from side-dependent topologically protected coupling of Janus source [24]. This selective coupling associated with the reactive power $\boldsymbol {\mathcal {R}}$ is based on its $\hat{T}$-symmetry and $\hat{P}$-antisymmetry, which can distinguish the branch pairs $\lbrace \boldsymbol {v}_1,\boldsymbol {v}_2\rbrace$ and $\lbrace \boldsymbol {v}_3,\boldsymbol {v}_4\rbrace$. Different from ‘two face’ properties of Janus source, the acoustic Huygens source, as shown in Fig. 3(f), can excite uni-directional surface modes because of the directional transport character of energy flow $\boldsymbol {\mathcal {J}}$. Huygens source can achieve the non-symmetric excitation along a single propagation direction, which reflects the $\hat{P}\hat{T}$ symmetry that can distinguish the branch pairs $\lbrace \boldsymbol {v}_1,\boldsymbol {v}_3\rbrace$ and $\lbrace \boldsymbol {v}_2,\boldsymbol {v}_4\rbrace$. The acoustic spin source, as shown in Fig. 3(g), will couple to the near-field modes in diagonal-paired directions. The selected couplings of the spin source result from ‘spin-momentum locking’ in longitudinal waves (see Method ‘Acoustic spin angular momentum’), which are universal for evanescent modes and reminiscent of the quantum spin Hall effect (QSHE) [22,23,26], independent of the transverse or longitudinal nature. This spin-momentum locking relationship reflects the $\hat{P}$-symmetry and $\hat{T}$-antisymmetry of the near-field acoustic SAM, which can distinguish the branch pairs $\lbrace \boldsymbol {v}_1,\boldsymbol {v}_4\rbrace$ and $\lbrace \boldsymbol {v}_2,\boldsymbol {v}_3\rbrace$.

**Figure 4.**
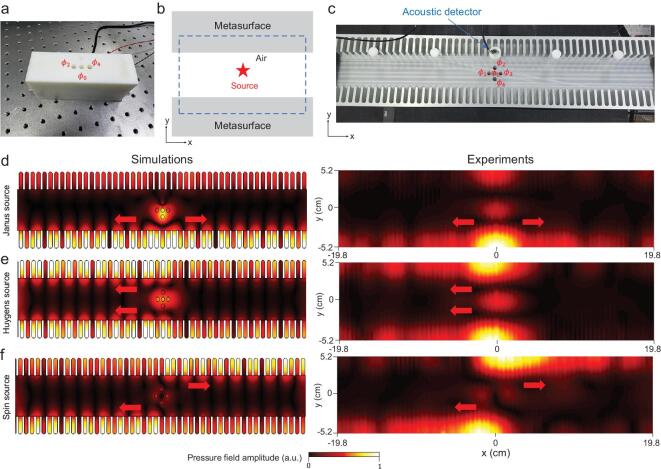
Experimental observations of the selective couplings of acoustic Janus, Huygens and spin sources. (a) Snapshot of Janus source made by the speaker array controlled by the external circuit. Huygens and spin sources are not shown here. (b) The detection areas of pressure field are shown in the blue dashed rectangle. (c) Photograph of the experimental setup. The comb-like aluminium structure is constructed as an acoustic meta-material with effective negative parameters, which thus supports fertile surface near-field acoustic wave modes. The numerical simulations and experimental results are shown, respectively: (d) Janus source, (e) Huygens source and (f) spin source. The experimental frequency is *f* = 2660 Hz. (More experimental details in Supplementary data.)

Now, we implement the experiments to verify the symmetry-selected directional near-field excitations, as shown in Fig. 4. The detection areas for the pressure field (blue dashed rectangle) are shown in Fig. 4(b). To support the near-field waveguide evanescent wave, comb-like structures (fabricated by metal aluminum) as shown in Fig. 4(c), were used because of their acoustic resonant structures [11,26,31–37]. For the Janus source $\boldsymbol {Q}_{\text{Janus}} = M - D_y$ formed by the field {φ_0_ = 1, φ_1_ = φ_3_ = 0, φ_4_ = −φ_2_ = 1} shown in Fig. 4(d), it will excite two branches of near-field acoustics satisfying $\hat{T}$-symmetry. For the Huygens source $\boldsymbol {Q}_{\text{Huygens}} = M - i D_x$ formed by the field {φ_0_ = 1, φ_1_ = −φ_3_ = −*i*, φ_4_ = φ_2_ = 0} shown in Fig. 4(e), it will excite two branches satisfying $\hat{P}\hat{T}$-symmetry. For the spin source $\boldsymbol {Q}_{\text{spin}} = D_x - i D_y$ formed by the field {φ_0_ = 0, φ_1_ = −φ_3_ = −*i*, φ_2_ = −φ_4_ = 1} shown in Fig. 4(f), it will excite two paired branches satisfying $\hat{P}$-symmetry. The experimental results are in good agreement with the theoretical analysis and numerical simulations. Because of the intrinsic thermoacoustic damping in actual experiments [38–40], observed surface waves will suffer from attenuations and have complex wave vectors. The amplitude of attenuations is related to the resonant strength of meta-structures [38–40]. Nevertheless, these selective excitation phenomena of our designed sources are still clearly observed, despite these loss effects.

**Figure 5.**
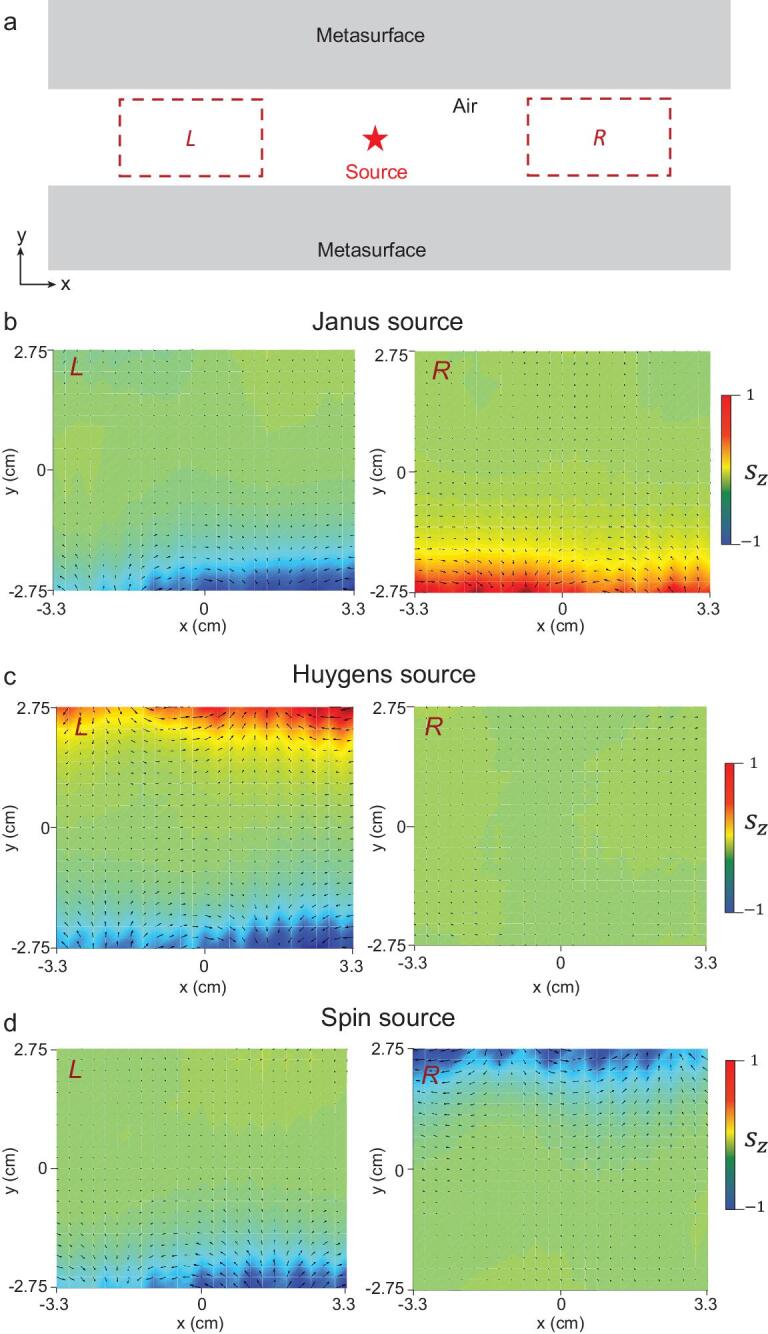
Experimental verifications of the symmetry of spin angular momentum in the acoustic near field of three different excitations. (a) The areas *L*\*R* (two red dashed rectangles) are the velocity detection regions. (b–d) The measured SAM densities of Janus, Huygens and spin sources with the same settings in Fig. 4 are plotted in the normalized colorbar. From the measured SAM densities, we can conclude that the near-field acoustic SAMs of the three sources have $\hat{T}$-symmetry, $\hat{P}\hat{T}$-symmetry and $\hat{P}$-symmetry, which are in good agreement with Fig. 2.

In addition to the observation of these directional phenomena, we measure the SAM density in the near-field acoustics and verify the symmetry properties experimentally. We couple the different sources of specific symmetries to the near-field modes with selected directionality. By exploiting the acoustic velocity sensor, we directly measure the amplitude and phase of the acoustic velocity field $v$_*x*_/$v$_*y*_ of the area shown in Fig. 5(a) and observe the circularly polarized velocity fields. Based on the definition of the SAM density $\boldsymbol {s} = \frac{\rho _0}{2\omega }{\rm Im}[\boldsymbol {v}^*\times \boldsymbol {v}]$, one can obtain the SAM density distribution. According to the results in Fig. 5(b–d), we can conclude that: the opposite SAM densities $\boldsymbol {s}$ for Janus source in different areas *L*\*R* reflect that acoustic SAM is $\hat{T}$-antisymmetric; the opposite $\boldsymbol {s}$ profiles for Huygens source excitations mean that the SAM is $\hat{P}\hat{T}$-antisymmetric; and the same $\boldsymbol {s}$ profiles for spin source excitations represent that the SAM is $\hat{P}$-symmetric. Moreover, we can see that the SAM is strongly associated with its momentum for the two $\hat{T}$-symmetric branches: the case of the Janus source in Fig. 5(b), which is also the experimental evidence for acoustic spin-momentum locking.

One more directional coupling case is to selectively couple the uni-directional one-side surface wave out of four branches. Symmetry breaking of all of them $\hat{P},\hat{T},\hat{P}\hat{T}$ could result in this effect and to achieve this goal, we need to take the acoustic quadrupole into account. Because the linear combinations of acoustic monopole and dipole for a point source can be rewritten as linear combinations of Janus, Huygens and spin sources, namely $\boldsymbol {Q}_s = c_1 \boldsymbol {Q}_{{\rm Janus}} + c_2 \boldsymbol {Q}_{{\rm Huygens}} + c_3 \boldsymbol {Q}_{{\rm Spin}}$, where *c*_*i*_ is the arbitary constant, and there is not such a combination {*c*_*i*_} that will result in excitations of uni-directional one-side surface wave out of four branches. The quadruple-related geometrical symmetry and topological characteristics have been explored and discussed in recent research works [41,42]. One can therefore realize one-side uni-directional excitation mainly based on spin-momentum locking and near-field interference between the spin source and quadruple one. This quadruple hybrid source will correspond to more complex geometrical conditions and the four different evanescent wave profiles in Fig. 1(b) will become distinguishable so as to be coupled independently (see Supplementary data).

Similar selective-directionality phenomena can be found in related optical works [24,28] and these similarities in near-fields are a consequence of the fundamental wave symmetry. We note that magnetic dipoles are still core components in these optical source realizations [24,28]. However, it should be mentioned that one can achieve alternative optical near-field sources without introducing magnetic dipoles. These near-field phenomena can be excited using only all electric dipoles or other flexible electric approaches with symmetry features, just following the design scheme we proposed in Fig. 3.

## CONCLUSION

To summarize, we have unveiled the inherent symmetry and geometric properties in near-field waves based on the acoustic wave platform. We have experimentally achieved efficient selective near-field wave coupling via acoustic Janus, Huygens and spin sources. The general physics behind these directional near-field couplings is based on their inherent geometry and symmetry-related mechanisms, not restricted to the special transverse wave system [23,24]. Our work could guide the directional design of promising applications of general near-field waves, especially for efficient coupling of wave energy transport, selective functions of acoustic/phononics devices, and new kinds of novel near-field sources. The symmetry-based source design approach could be generalized into other wave research fields, for example quantum optics [43] and elastic waves [25].

## METHODS

### The coupling theory of acoustic sources

In this section, we will show how to calculate the coupling strength *C*. The acoustic wave equation can be described as using the following equations:

(6) \begin{eqnarray*} &&\nabla p + \rho _0 \frac{\partial }{\partial t}\boldsymbol {v} = 0\nonumber\\ &&\nabla \cdot \boldsymbol {v} + \frac{1}{\rho _0 c^2} \frac{\partial }{\partial t}p = 0, \end{eqnarray*}

where *p* is the pressure, ρ_0_ is mass density, *c* is the acoustic speed, and $\boldsymbol {v}$ is the velocity. To unveil the far-field radiations and near-field couplings of acoustic sources, the acoustic wave equation can be written in the following form:

(7) \begin{eqnarray*} &&\nabla ^2\boldsymbol {v} - \frac{1}{c^2}\frac{\partial ^2}{\partial t^2}\boldsymbol {v} = -\boldsymbol {Q}_v \nonumber\\ &&\nabla ^2 p - \frac{1}{c^2}\frac{\partial ^2}{\partial t^2}p = -Q_p, \end{eqnarray*}

where the pressure *p* and velocity $\boldsymbol {v}$ satisfy the relation $\nabla \cdot \boldsymbol {v} + \frac{1}{\rho _0 c^2}\frac{\partial }{\partial t}p=0$, which has a similar form to the Lorentz gauge for electromagnetic wave. Eq. (7) has the same forms as D’Alembert’s equations, which describe the radiation and excitation of electromagnetic waves. The terms $\lbrace \boldsymbol {Q}_v,Q_p\rbrace$ are the acoustic sources.

Introducing the source vector $\mathcal {E} = (Q_{vx}, Q_{vy},Q_{vz},Q_p)$ and the wave descriptor vector $\mathcal {F} = (v_x,v_y,v_z,-\frac{i}{\rho _0 c}p)$, we can thus calculate the coupling coefficient *C* based on the Fermi golden rule:

(8) \begin{eqnarray*} C \sim \mathcal {F}^*\cdot \mathcal {E} &=& v_x^*Q_{vx} + v_y^*Q_{vy}\nonumber\\ +\, v_z^*Q_{vz} + \frac{i}{\rho _0 c}p^*Q_p. \end{eqnarray*}

The |*C*|^2^ will unveil the strength of coupling and predict the radiation patterns and couplings of sources. The source $\boldsymbol {Q}_s = \alpha M + \boldsymbol {\beta }\cdot \boldsymbol {D}$ will result in the source vector:

(9) \begin{equation*} \mathcal {E} = (\beta _x,\beta _y,\beta _z,\alpha). \end{equation*}

For the cases in Fig. 1, their pressure fields and velocity fields have evanescent forms as:

(10) \begin{eqnarray*} p &=& p_0 e^{i (k x-\omega t) - \tau y} \nonumber\\ \boldsymbol {v} &=& \frac{p_0}{\rho _0 \omega } \left(\begin{array}{c}k \\ i\tau \\ 0 \end{array}\right) e^{i (k x-\omega t) - \tau y}. \end{eqnarray*}

And thus, their corresponding wave descriptor vector can be written as:

(11) \begin{eqnarray*} \mathcal {F} &=& p_0 \left(\frac{k}{\rho _0 \omega }, \frac{i\tau }{\rho _0 \omega }, 0, -\frac{i}{\rho _0 c} \right) \nonumber\\ &=&\frac{p_0}{\rho _0 \omega } (k, i\tau , 0, -i k_0)\nonumber\\ &\sim & (k, i\tau , 0, -i k_0), \end{eqnarray*}

where $k_0 = \frac{\omega }{c}$.

Janus source has the form *M* ± *D*_*y*_, so its near-field coupling coefficient can be calculated as:

(12) \begin{eqnarray*} C &\sim & \mathcal {F}^*\cdot \mathcal {E} \nonumber\\ &=& (k, i\tau , 0, -ik_0)^*\cdot (0,\pm 1,0,1) \nonumber\\ &=& i ( k_0 \mp \tau ), \end{eqnarray*}

where |*C*|^2^ ∼ |*i*(*k*_0_ ∓ τ)|^2^ = (*k*_0_ ∓ τ)^2^.

Huygens source has the form *M* ± *iD*_*x*_, so its near-field coupling coefficient can be calculated as:

(13) \begin{eqnarray*} C &\sim & \mathcal {F}^*\cdot \mathcal {E} \nonumber\\ &=& (k, i\tau , 0, -i k_0)^*\cdot (\pm i,0,0,1) \nonumber\\ &=& i (k_0 \pm k), \end{eqnarray*}

where |*C*|^2^ ∼ |*i*(*k*_0_ ± *k*)|^2^ = (*k*_0_ ± *k*)^2^.

Spin source has the form *D*_*x*_ ± *iD*_*y*_, so its near-field coupling coefficient can be calculated as:

(14) \begin{eqnarray*} C &\sim &\mathcal {F}^*\cdot \mathcal {E} \nonumber\\ &=& (k, i\tau , 0, i k_0)^*\cdot (1, \pm i,0,0) \nonumber\\ &=& k \pm \tau , \end{eqnarray*}

where |*C*|^2^ ∼ |*k* ± τ|^2^ = (*k* ± τ)^2^.

Thus the near-field couplings of these sources can be written as:

(15) \begin{eqnarray*} |C_{{\rm Janus}}|^2 &\sim & (k_0 \mp \tau )^2 \nonumber\\ |C_{{\rm Huygens}}|^2 &\sim & (k_0 \pm k)^2 \nonumber\\ |C_{{\rm Spin}}|^2 &\sim & (k \pm \tau )^2 = k^2\left(1\pm \frac{\tau }{k}\right)^2. \nonumber\\ \end{eqnarray*}

From the result, we can see that the Janus source excites the evanescent wave based on the decay rate τ, which reflects the reactive power $\propto {\rm Im}[p\boldsymbol {v}^*]$. The Huygens source excites the evanescent wave based on the wave vector *k*, which is associated with the time-averaged energy flow $\propto {\rm Re}[p\boldsymbol {v}^*]$. The spin source excites the evanescent wave based on the ratio τ/*k*, which can be described by acoustic SAM density $\boldsymbol {s} \propto {\rm Im}[\boldsymbol {v}^*\times \boldsymbol {v}]$. Moreover from these calculated results, we can see that the near-field couplings of sources could be totally different from their far-field patterns (see Supplementary data).

### Acoustic spin angular momentum

In this section, we provide details on the spin angular momentum of near-field acoustics. For the acoustic wave, the acoustic momentum can be described by the time average Poynting momentum vector $\boldsymbol {\mathcal {P}}=\frac{1}{2c^2}{\rm Re}[p^*\boldsymbol {v}]$, which can be called the momentum density of acoustic wave. It is well known that the momentum density $\boldsymbol {\mathcal {P}}$ can be expressed as the expectation value of momentum density operator $\hat{\boldsymbol {p}}$ in quantum mechanics. For harmonic acoustic wave $\boldsymbol {v}(\boldsymbol {r},t) = \boldsymbol {v}(\boldsymbol {r})e^{-i\omega t}$, the momentum density $\boldsymbol {\mathcal {P}}$ can be written as:

(16) \begin{eqnarray*} \boldsymbol {\mathcal {P}} &=& \frac{1}{2c^2}{\rm Re}\left[\frac{i\rho _0 c^2}{\omega } (\nabla \cdot \boldsymbol {v}^*)\boldsymbol {v}\right]\nonumber\\ & =& \frac{\rho _0}{2\omega }{\rm Im}\left[(\nabla \cdot \boldsymbol {v})\boldsymbol {v}^*\right], \end{eqnarray*}

with the relation $p = -\frac{i\rho _0 c^2}{\omega } \nabla \cdot \boldsymbol {v}$, ρ_0_ is the mass density. With mathematical expansion ${\rm Im}[(\nabla \cdot \boldsymbol {v})\boldsymbol {v}^*]\, = \,{\rm Im}[\boldsymbol {v}^*\cdot (\nabla )\boldsymbol {v}]\, +\, \frac{1}{2}{\rm Im}[\nabla \times (\boldsymbol {v}^*\times \boldsymbol {v})]$, the momentum density $\boldsymbol {\mathcal {P}}$ can be regarded as the combination of two parts with different physical meanings $\boldsymbol {\mathcal {P}} = \boldsymbol {\mathcal {P}}^o + \boldsymbol {\mathcal {P}}^s$, where $\boldsymbol {\mathcal {P}}^o$ and $\boldsymbol {\mathcal {P}}^s$ are orbital and spin momentum density, respectively [22,25,26,44]. For acoustic waves, these two terms will have the following forms:

(17) \begin{eqnarray*} \boldsymbol {\mathcal {P}}^o &=& \frac{\rho _0}{2\omega }{\rm Im}[\boldsymbol {v}^*\cdot (\nabla )\boldsymbol {v}] \nonumber\\ \boldsymbol {\mathcal {P}}^s &=& \frac{\rho _0}{4\omega }\nabla \times {\rm Im}[\boldsymbol {v}^*\times \boldsymbol {v}]. \end{eqnarray*}

The spin momentum density of acoustic waves can be regarded as the curl of spin angular momentum density, $\boldsymbol {\mathcal {P}}^s = \frac{1}{2}\nabla \times \boldsymbol {s}$ [45]. The spin angular momentum density $\boldsymbol {s}$ for acoustics will be [25–27]:

(18) \begin{equation*} \boldsymbol {s} = \frac{\rho _0}{2\omega }{\rm Im}[\boldsymbol {v}^*\times \boldsymbol {v}]. \end{equation*}

Alternatively, the time-averaged acoustic angular momentum $\boldsymbol {AM}$ can be separated as:

(19) \begin{eqnarray*} \boldsymbol {AM} & =& \int \left\langle \boldsymbol {r}\times \rho \boldsymbol {v} \right\rangle _t d\!\boldsymbol {r}^3\nonumber\\ & =& \frac{\rho _0}{2\omega } \int \boldsymbol {r}\times {\rm Im}[(\nabla \cdot \boldsymbol {v})\boldsymbol {v}^*] d\!\boldsymbol {r}^3 \nonumber\\ &=&\frac{\rho _0}{2\omega } \int \boldsymbol {r} \times \bigg({\rm Im}[\boldsymbol {v}^*\cdot (\nabla )\boldsymbol {v}]\nonumber\\ +\, \frac{1}{2}\nabla \times {\rm Im}[\boldsymbol {v}^*\times \boldsymbol {v}]\bigg) d\!\boldsymbol {r}^3 \nonumber\\ &=& \frac{\rho _0}{2\omega } \int \boldsymbol {r}\times {\rm Re} [\boldsymbol {v}^*\cdot (-i\nabla )\boldsymbol {v}] d\!\boldsymbol {r}^3\nonumber\\ +\, \frac{\rho _0}{2\omega } \int {\rm Im}[\boldsymbol {v}^*\times \boldsymbol {v}] d\!\boldsymbol {r}^3 \nonumber\\ &=&\boldsymbol {L} + \boldsymbol {S}, \end{eqnarray*}

where $\boldsymbol {r}$ is the position vector, 〈·〉_*t*_ is the time-averaged operation, and the mass continuum condition $\partial _t \rho + \rho _0 (\nabla \cdot \boldsymbol {\boldsymbol {v}}) = 0$ has been used. $\boldsymbol {S} = \int \boldsymbol{s}\!d\!\boldsymbol{r}^3$ is the SAM, with density $\boldsymbol {s}= \frac{\rho _0}{2\omega } {\rm Im}[\boldsymbol {v}^*\times \boldsymbol {v}]$. $\boldsymbol {L} = \int \boldsymbol{l}\!d\!\boldsymbol {r}^3$ is the OAM with density $\boldsymbol {l}= \frac{\rho _0}{2\omega }{\rm Re}[\boldsymbol {v}^*\cdot (-i\!\boldsymbol{r}\times \nabla )\boldsymbol {v}]=\boldsymbol {r}\times \boldsymbol {\mathcal {P}}^o$.

Introducing the state vector about acoustic field, $|\boldsymbol {v}\rangle = \sqrt{\frac{\rho _0}{2\omega }}(v_x,v_y,v_z)^T$, one can represent the spin angular momentum density in concrete and clear quantum form:

(20) \begin{equation*} \boldsymbol {s} =\langle \boldsymbol {v}|\hat{\boldsymbol {S}}|\boldsymbol {v}\rangle , \end{equation*}

where $\boldsymbol {v}^*\cdot (\hat{\boldsymbol {S}})\boldsymbol {v}= {\rm Im}[\boldsymbol {v}^*\times \boldsymbol {v}]$ is the spin angular momentum density operator. The spin angular momentum density of acoustic wave can be described by taking the following spin-1 operator on state vector $|\boldsymbol {v}\rangle$:

(21) \begin{eqnarray*} \hat{S}_{x} &=& {\left(\begin{array}{ccc}0 &\quad 0 &\quad 0\\ 0 &\quad 0 &\quad -i\\ 0 &\quad i &\quad 0 \end{array}\right)}, \hat{S}_{y} = {\left(\begin{array}{ccc}0 &\quad 0 &\quad i\\ 0 &\quad 0 &\quad 0\\ -i &\quad 0 &\quad 0 \end{array}\right)},\nonumber\\ \hat{S}_{z} &=& {\left(\begin{array}{ccc}0 &\quad -i &\quad 0\\ i &\quad 0 &\quad 0\\ 0 &\quad 0 &\quad 0 \end{array}\right)}, \end{eqnarray*}

where $\langle \boldsymbol {v}|\hat{\boldsymbol {S}}|\boldsymbol {v}\rangle \propto {\rm Im}[\boldsymbol {v}^*\times \boldsymbol {v}]$. The spin operator $\hat{\boldsymbol {S}}$ satisfies the fundamental commutation relations of angular momentum: [*S*_*i*_, *S*_*j*_] = *i*ε_*ijk*_*S*_*k*_.

For Fig. 2, the surface mode can be described as (set *y* = 0 as the interface between air and effective media):

(22) \begin{eqnarray*} \boldsymbol {v}_1 &=& \frac{p_0}{\rho _1 \omega } {\left(\begin{array}{c}k \\ i \tau _1 \\ 0 \\ \end{array}\right)} e^{i (k x- \omega t)-\tau _1 y} \nonumber\\ \boldsymbol {v}_2 &=& \frac{p_0}{\rho _2 \omega } {\left(\begin{array}{c}k \\ -i \tau _2 \\ 0 \\ \end{array}\right)} e^{i (k x- \omega t)+\tau _2 y}, \end{eqnarray*}

where $\boldsymbol {v}_1$ and $\boldsymbol {v}_2$ are the velocity field for air and media, respectively, *c*_1_ and *c*_2_ are the acoustic speeds, ρ_1_ and ρ_2_ are the mass densities, specially ρ_2_ < 0, $k^2 - \tau _1^2 = \frac{\omega ^2}{c_1^2}$ and $k^2-\tau _2^2=\frac{\omega ^2}{c_2^2}$, *k* is wave vector. The acoustic wave satisfies the dispersion relationship : $k^2 = \frac{c_2^2\rho _2^2-c_1^2\rho _1^2}{c_1^2 c_2^2 (\rho _2^2-\rho _1^2)}\omega ^2$, which could be obtained by considering boundary conditions on the interface. According to the acoustic spin, the spin density of the corresponding acoustic surface mode can be represented as:

(23) \begin{eqnarray*} \boldsymbol {s}_1 &=& \langle \boldsymbol {v}_1|\hat{\boldsymbol {S}}|\boldsymbol {v}_1\rangle = \frac{c_1^2 p_0^2}{2\rho _1 \omega ^3} 2\tau _1 k e^{-2 \tau _1 y} \boldsymbol {e}_z\nonumber\\ \boldsymbol {s}_2 &=& \langle \boldsymbol {v}_2|\hat{\boldsymbol {S}}|\boldsymbol {v}_2\rangle = -\frac{c_2^2 p_0^2}{2\rho _2 \omega ^3} 2\tau _2 k e^{2 \tau _2 y} \boldsymbol {e}_z . \end{eqnarray*}

From Eq. (23), we find that $\boldsymbol {s} \propto k\tau \boldsymbol {e}_z$, which reflects spin-momentum locking of surface wave. From the calculated $\boldsymbol {s}$, the sign of total spin density $\boldsymbol {S} = \int \boldsymbol {s}\! d\!y$ will become opposite for different wave vector *k*: *S*_$z$_ > 0 for *k* > 0, *S*_$z$_ < 0 for *k* < 0, as shown in Fig.S5 of the Supplementary data, which has similar properties to that in quantum spin Hall effect (QSHE) [46] and 3D topological insulators for electrons [47]. Thus this spin momentum locking phenomena can be seen as the QSHE of the acoustic wave [26,27]. Similar to the QSHE of light [22], these modes cannot be immune with backscattering induced by impurity and disorder without time-reversal broken.

Based on the acoustic SAM, $\frac{\tau }{k}=\frac{\tau k}{k^2} \propto \frac{1}{k^2}\boldsymbol {s}\cdot \boldsymbol {e}_z$, the near-field excitation of the spin source in Eq. (15) can be written as:

(24) \begin{equation*} |C_{{\rm spin}}|^2 \propto \left(1 \pm \frac{1}{k^2} \boldsymbol {s}\cdot \boldsymbol {e}_z\right)^2. \end{equation*}

The near-field coupling strength of the spin source can be associated with the acoustic SAM density carried in the evanescent wave. Obviously, this coupling is strongly locked with the sign of acoustic SAM.

### Experimental proposal for arbitrary acoustic sources

In this section, we will supply one feasible experimental scheme to achieve a general near-field acoustic source, and give a practical example to realize Janus, Huygens and spin sources.

**Figure 6.**
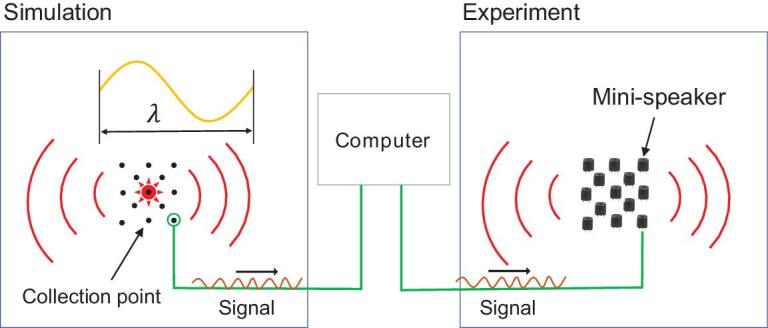
Feasible experimental scheme to realize the general near-field acoustic source. The designed speaker array can be regarded as an acoustic meta-source because of its subwavelength scale.

A scheme to achieve a general near-field acoustic source is shown in Fig. 6. Firstly, in numerical simulations, we can set several collection points surrounding the theoretic general acoustic source compactly, in smaller-than-wavelength scale (<17 cm for 2 kHz), to record the strength and phase of the acoustic near-field. Secondly, we can place the acoustic speakers at the corresponding positions of these collection points. Thirdly, we play these speakers (can be regarded as monopole) with the strength and phase recorded by the corresponding collection points. This scheme is based on Huygens principle in optics. The number and spatial distribution of collection points or speakers depends on the practical engineering requirements and working frequency. Because the scale of the designed speaker array is smaller than the wavelength in air, this speaker array can be regarded as an acoustic meta-source. This meta-source can be functional to achieve any combination of acoustic monopole, dipoles and quadrupoles.

To exemplify one experimental scheme, we will exploit speaker array to realize the Janus, Huygens and spin sources in the main text. The physical mechanism of these sources can be understood as a linear combination of different kinds of basic acoustic sources $\boldsymbol {v}_s = \alpha M + \beta _x D_x + \beta _y D_y$ and the coefficient set α, β_*x*_, β_*y*_ will be responsible for achieving these sources. Thus, we can design such a source device shown in Fig. 3, in which the five speakers are placed in a cross bending shape and excited with specific strength and phase settings. The center speaker φ_0_ plays the role of acoustic monopole *M*. The other four speakers φ_1_, φ_2_, φ_3_, φ_4_ will behave as effective acoustic dipoles *D*_*x*_, *D*_*y*_ when they are set as:

(25) \begin{eqnarray*} \phi _1 &=& - \phi _3 \rightarrow D_x \nonumber\\ \phi _2 &=& - \phi _4 \rightarrow D_y. \end{eqnarray*}

Thus, it’s easy to construct Janus, Huygens and spin sources as:

(26) \begin{eqnarray*} &&{\rm Janus \,\, source}\!\!:\nonumber\\ &&(\phi _0,\phi _1,\phi _2,\phi _3,\phi _4) =(1,0, \pm 1, 0, \mp 1) \nonumber\\ &&{\rm Huygens \,\, source}\!\!:\nonumber\\ &&(\phi _0,\phi _1,\phi _2,\phi _3,\phi _4) =(1,\pm i, 0, \mp i,0) \nonumber\\ &&{\rm Spin \,\, source}\!\!:\nonumber\\ &&(\phi _0,\phi _1,\phi _2,\phi _3,\phi _4) =(0, 1, \pm i, -1, \mp i). \nonumber\\ \end{eqnarray*}

## Supplementary Material

nwaa040_Supplemental_File
