# Supplementary material for: Symmetry selective directionality in near-field acoustics
Source: Natl Sci Rev. 2020 Mar 14;7(6):1024–35. doi: 10.1093/nsr/nwaa040 (PMC8289114; doi:10.1093/nsr/nwaa040)
Supplement: nwaa040_Supplemental_File [file nwaa040_supplemental_file.pdf]

Symmetry Selective Directionality in Near-Field Acoustics - Supplementary

Yang Long[1,*] Hao Ge[2,*] Danmei Zhang[1], Xiangyuan Xu[2,3], Jie Ren[1,†]
Ming-Hui Lu[2,4,‡] Ming Bao[3], Hong Chen[1], and Yan-Feng Chen[2,4]
[1] *Center for Phononics and Thermal Energy Science, China-EU Joint Center for Nanophononics, Shanghai Key Laboratory of Special Artificial Microstructure Materials and Technology, School of Physics Sciences and Engineering, Tongji University, Shanghai 200092, China*
[2] *Department of Materials Science and Engineering, College of Engineering and Applied Sciences and National Laboratory of Solid State Microstructures, Nanjing University, Nanjing 210093, China*
[3] *Key Laboratory of Noise and Vibration Research, Institute of Acoustics, Chinese Academy of Sciences, Beijing 100190, China*
[4] *Collaborative Innovation Center of Advanced Microstructures, Nanjing University, Nanjing, Jiangsu 210093, China*
(Dated: December 16, 2019)

FAR-FIELD RADIATION PATTERN BEHAVIOUR

Considering the harmonic acoustic wave as:

$$p = p_0 e^{i(k_x x + k_y y + k_z z - \omega t)} \tag{S1}$$

one can get its velocity field as:

$$\boldsymbol{v} = \frac{p_0}{\rho\omega}\begin{pmatrix} k_x \\ k_y \\ k_z \end{pmatrix} e^{i(k_x x + k_y y + k_z z - \omega t)} \tag{S2}$$

Thus, the corresponding wave descriptor vector can be written as:

$$\begin{aligned} \mathcal{F} &= \left(v_x, v_y, v_z, -\frac{i}{\rho c} p\right) \\ &= p_0 \left(\frac{k_x}{\rho\omega}, \frac{k_y}{\rho\omega}, \frac{k_z}{\rho\omega}, -\frac{i}{\rho c}\right) \\ &= \frac{p_0}{\rho\omega}(k_x, k_y, k_z, -ik_0) \\ &\sim (k_x, k_y, k_z, -ik_0) \end{aligned} \tag{S3}$$

where $k_0 = \frac{\omega}{c}$. Here, we focus on the far field radiation on xOy plane, which will result $\mathcal{F} \sim k_0 (\cos(\theta), \sin(\theta), 0, -i)$, θ is the angle.

Janus source has the form $M \pm D_y$, so its far field coupling coefficient can be calculated as:

$$\begin{aligned} C &\sim \mathcal{F}^* \cdot \mathcal{E} \\ &= (\cos(\theta), \sin(\theta), 0, -i)^* \cdot (0, \pm 1, 0, 1) \\ &= i \pm \sin(\theta) \end{aligned} \tag{S4}$$

which results $|C|^2 \sim |i \pm \sin(\theta)|^2 = 1 + \sin^2(\theta)$.

Huygens source has the form $M \pm iD_x$, so its far field coupling coefficient can be calculated as:

$$\begin{aligned} C &\sim \mathcal{F}^* \cdot \mathcal{E} \\ &= (\cos(\theta), \sin(\theta), 0, -i)^* \cdot (\pm i, 0, 0, 1) \\ &= i \pm i\cos(\theta) \end{aligned} \tag{S5}$$

which results $|C|^2 \sim |i \pm i\cos(\theta)|^2 = (1 \pm \cos(\theta))^2$.

Spin source has the form $D_x \pm iD_y$, so its far field coupling coefficient can be calculated as:

$$\begin{aligned} C &\sim \mathcal{F}^* \cdot \mathcal{E} \\ &= (\cos(\theta), \sin(\theta), 0, -i)^* \cdot (1, \pm i, 0, 0) \\ &= \cos(\theta) \pm i\sin(\theta) \end{aligned} \tag{S6}$$

which results $|C|^2 \sim |\cos(\theta) \pm i\sin(\theta)|^2 = \cos^2(\theta) + \sin^2(\theta) = 1$.

Thus the far field radiation patterns of these sources can be written as:

$$\begin{aligned} |C^{\text{far}}_{\text{Janus}}|^2 &\sim 1 + \sin^2(\theta) \\ |C^{\text{far}}_{\text{Huygens}}|^2 &\sim (1 \pm \cos(\theta))^2 \\ |C^{\text{far}}_{\text{Spin}}|^2 &\sim 1 \end{aligned} \tag{S7}$$

To verify the theoretical analysis of the far field pattern of these sources, the numerical results have been calculated and compared with theoretical results shown in Fig.S1. The calculated far field patterns, the insets in Fig.S1, have shown that the coupling strength can reflect the radiation properties of sources well.

UNI-DIRECTIONAL RADIATION OF ACOUSTIC WAVE BEAM IN BULK

With help of acoustic Huygens source, we can realize uni-directional excitation of acoustic wave beam by utilizing the Huygens sources array. The radiation direction can be controlled by adjusting phase gradient ϕ, shown in Fig.S2(a), which can be represented as:

$$\boldsymbol{Q}_s = \sum_n \alpha_0 e^{in\phi} \delta(\boldsymbol{r} - \boldsymbol{r}_n)(M \pm iD_x) \tag{S8}$$

where δ is delta function, $\boldsymbol{r}_n$ is the position of the n-th Huygens dipole, α_0 is the amplitude constant. The physical mechanism of uni-directional wave beam excitation is similar with the phase array antenna in microwave engineering. The numerical results shown in Fig.S2(b) show that the Huygens sources array can be exploited to excite uni-directional acoustic wave beam. The direction of wave beam can be controlled by adjusting the phase gradient ϕ.

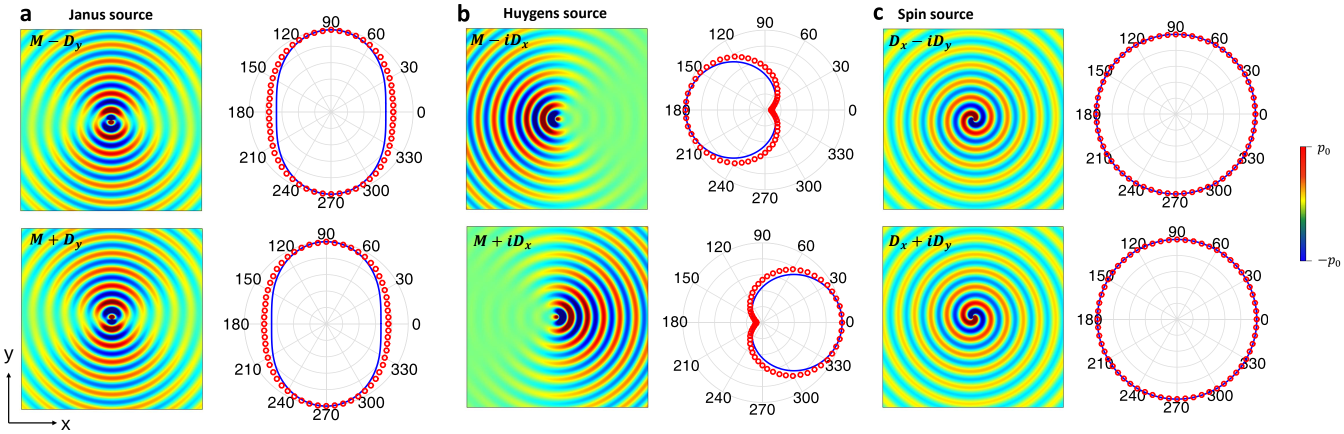

FIG. S1. The far field radiation pattern of Janus, Huygens and spin sources. (a)Janus source. (b)Huygens source. (c) Spin source. In their far field patterns, the theoretical and numerical results are shown in blue lines and red circles respectively.

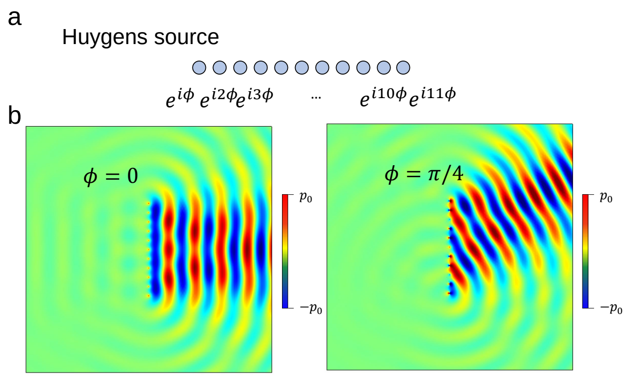

FIG. S2. Uni-directional excitation of acoustic wave beam. (a) The Huygens sources array with excitation phase gradient ϕ. (b) The direction of excited wave beam can be controlled by phase gradient ϕ.

UNI-DIRECTIONAL ONE-SIDE EXCITATION WITH QUADRUPOLE

One more intriguing case is to excite uni-directional one-side surface wave selectively. The symmetry breaking could result this effect. However, the linear combinations of acoustic monopole and dipole would alway conserve at least one symmetry due to the geometrical limit of monopole and dipole. To break several symmetry simultaneously, we should take the acoustic quadrupole into account: Quardrupole hybird source: $\boldsymbol{Q}_s = \beta_x D_x + \beta_y D_y + \gamma_{xx} T_{xx} + \gamma_{xy} T_{xy} + \gamma_{yx} T_{yx} + \gamma_{yy} T_{yy}$. The quadruple-related geometrical symmetry and topological characteristics have been explored and discussed in recent research works [1, 2]. For quadrupole, it is associated with the high-order spatial gradient of pressure field and in xOy plane, its source vector $\mathcal{E}$ can be written as :

$$\mathcal{E} = \begin{pmatrix} 0 \\ 0 \\ 0 \\ \gamma_{xx}\frac{\partial^2}{\partial x^2}p^* + \gamma_{xy}\frac{\partial^2}{\partial x \partial y}p^* + \gamma_{yx}\frac{\partial^2}{\partial y \partial x}p^* + \gamma_{yy}\frac{\partial^2}{\partial y^2}p^* \end{pmatrix} \tag{S9}$$

where $\{\gamma\}$ represent its geometrical properties.

To realized the uni-directional one-side near field coupling, we exploit the quadrupole hybrid source as:

$$\mathcal{E} \sim \begin{pmatrix} \beta_x \\ \beta_y \\ 0 \\ \gamma_{yx}\frac{\partial^2}{\partial y \partial x}p^* + \gamma_{yy}\frac{\partial^2}{\partial y^2}p^* \end{pmatrix} \tag{S10}$$

For the harmonic far field wave form, it will result:

$$\mathcal{E} \sim \begin{pmatrix} \beta_x \\ \beta_y \\ 0 \\ -\gamma_{yx}k_0^2\cos(\theta)\sin(\theta) - \gamma_{yy}k_0^2\sin^2(\theta) \end{pmatrix} \tag{S11}$$

For the near field wave form, it will result:

$$\mathcal{E} \sim \begin{pmatrix} \beta_x \\ \beta_y \\ 0 \\ \gamma_{yx}ik\tau + \gamma_{yy}\tau^2 \end{pmatrix} \tag{S12}$$

Here, we focus one case to discuss its far field radiation pattern and near field excitation, $\beta_x = 1$, $\beta_y = i$, $\gamma_{yx} = \gamma_0$, $\gamma_{yy} = i\gamma_0$, where $\gamma_0 > 0$ is real constant to adjust field strength of quadrupole into the same scale of dipoles.

For far field radiation in xOy plane, its coupling coefficient

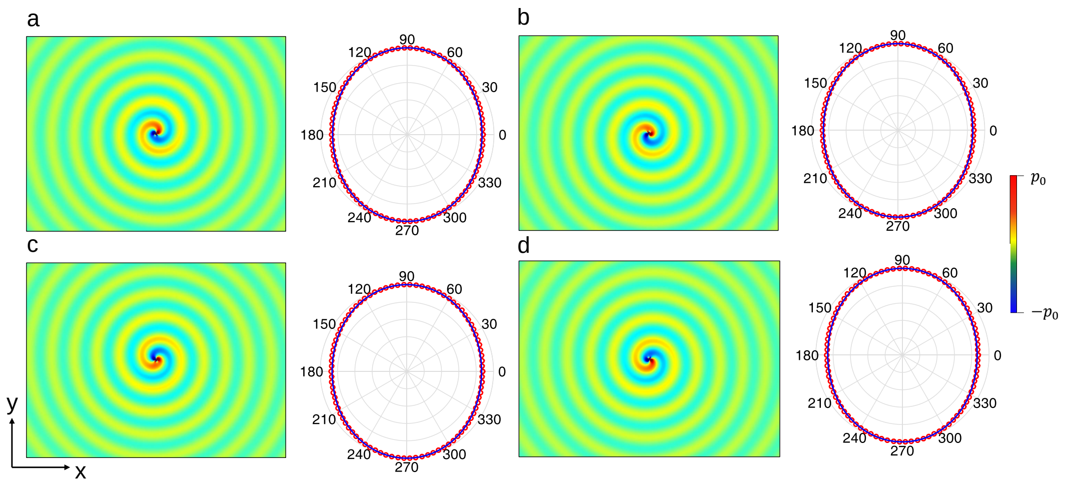

FIG. S3. The far field radiation pattern of quadrupole hybrid source. (a) $\beta_x = 1$, $\beta_y = i$, $\gamma_{yx} = \gamma_0$, $\gamma_{yy} = i\gamma_0$. (b) $\beta_x = 1$, $\beta_y = -i$, $\gamma_{yx} = \gamma_0$, $\gamma_{yy} = -i\gamma_0$. (c) $\beta_x = 1$, $\beta_y = -i$, $\gamma_{yx} = -\gamma_0$, $\gamma_{yy} = i\gamma_0$. (d) $\beta_x = 1$, $\beta_y = i$, $\gamma_{yx} = -\gamma_0$, $\gamma_{yy} = -i\gamma_0$. The $\gamma_0 > 0$ is real constant to adjust field strength of quadrupole into the same scale of dipoles. In their far field patterns, the theoretical and numerical results are shown in blue lines and red circles respectively.

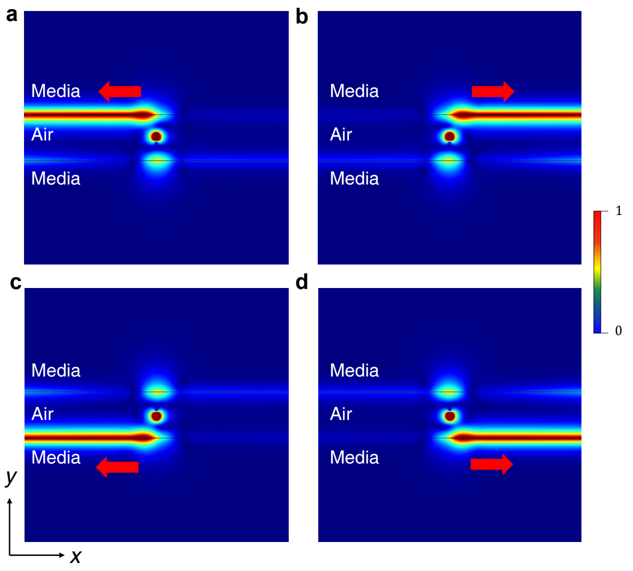

FIG. S4. Uni-directional one-side excitation of quadrupole hybrid source. After considering acoustic quadrupole, several geometric symmetries can be broken simultaneously. Different linear combinations have been calculated. (d) $\beta_x = 1$, $\beta_y = i$, $\gamma_{yx} = \gamma_0$, $\gamma_{yy} = i\gamma_0$. (e) $\beta_x = 1$, $\beta_y = -i$, $\gamma_{yx} = \gamma_0$, $\gamma_{yy} = -i\gamma_0$. (f) $\beta_x = 1$, $\beta_y = -i$, $\gamma_{yx} = -\gamma_0$, $\gamma_{yy} = i\gamma_0$. (g) $\beta_x = 1$, $\beta_y = i$, $\gamma_{yx} = -\gamma_0$, $\gamma_{yy} = -i\gamma_0$. The $\gamma_0 > 0$ is real constant to adjust field strength of quadrupole into the same scale of dipole. The pressure field amplitudes have been plotted. Here, we set $\gamma_{xx} = \gamma_{xy} = 0$ for all cases.

can be described as:

$$\begin{aligned} C &\sim \mathcal{F}^* \cdot \mathcal{E} \\ &= (\cos(\theta), \sin(\theta), 0, -i)^* \\ &\cdot \left(1, i, 0, \gamma_0 k_0^2(-\cos(\theta)\sin(\theta) - i\sin^2(\theta)\right) \\ &= \cos(\theta) + i\sin(\theta) - i\gamma_0 k_0^2(\cos(\theta)\sin(\theta) + i\sin^2(\theta)) \end{aligned} \tag{S13}$$

the coupling strength $|C|^2$ have been plotted in the inset of Fig.S3. From the results, we can see that the coupling theory work well for quadrupole hybrid sources.

For near field excitation, its coupling coefficient can be described as:

$$\begin{aligned} C &\sim \mathcal{F}^* \cdot \mathcal{E} \\ &= (k, i\tau, 0, -ik_0)^* \cdot (1, i, 0, \gamma_0(ik\tau + \tau^2)) \\ &= (k + \tau)(1 - \gamma_0 k_0 \tau) \end{aligned} \tag{S14}$$

From the $|C|^2 \sim |(k + \tau)(1 - \gamma_0 k_0 \tau)|^2$, we can see that $|C|^2 \approx 0$ when $\tau > 0$ or $k * \tau < 0$, which means this quadrupole hybrid source only couples uni-direction one-side surface mode ($\tau < 0$ and $k < 0$) strongly (corresponding to the case in Fig.5(a) of main text). For other forms of quadrupole hybrid source, their coupling strengths have similar form: $|C|^2 \sim |(k \pm \tau)(1 \pm \gamma_0 k_0 \tau)|^2$.

The simulation results in Fig. S4 show that the linear combination of dipoles and quadrupole will achieve uni-directional one-side excitation of surface acoustic waves out of four branches. The physical reason behind these one-side uni-directional couplings in Fig. S4 is mainly based on the spin-momentum locking and near field interference between spin sources and quadruple. The evanescent wave forms in Fig.1(c) of main text will become distinguishable to be coupled independently.

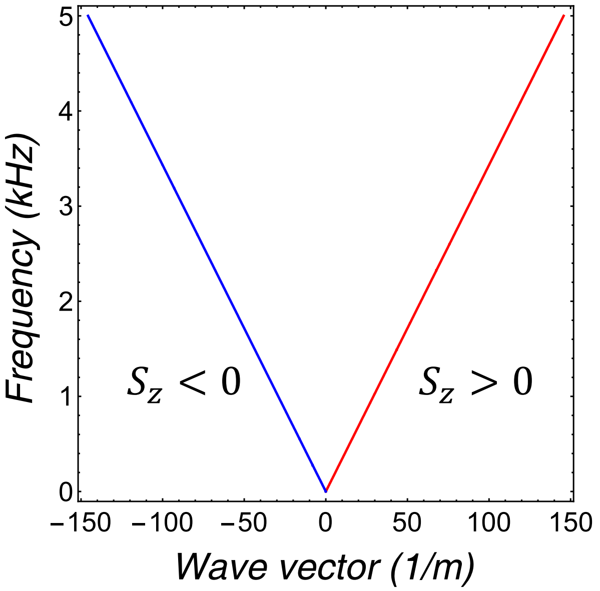

FIG. S5. The dispersion relationship of surface mode and spin-momentum locking of acoustic wave. The surface mode can be supported on the interface between air and media, discussed in main text. The acoustic spin is locked with momentum, $\boldsymbol{S} \propto k\boldsymbol{e}_z$.

EXPERIMENTAL DETAILS

The sample is fabricated by with the metal aluminum, which can be treated as acoustically rigid. One velocity field detector has been exploited to measure the acoustic velocity field and the detected velocity field is associated with the detector geometrical setting, shown in Fig. S6(a). The excitation amplitude and phase of speakers in Fig. S6(b) are controlled by the external circuit system. The sample is covered by an acrylic plate, shown in Fig. S6(c), and there is a tiny air gap (with a thickness less than 1mm) between the sample and the plate. For detecting the pressure field, an 1/4-inch microphone (GRAS Type 46BE) is placed inside the hole of the acrylic plate to detect the sound fields below the plate. The detected signals are acquired by the NI 9234 data acquisition module. The loudspeakers are driven by the NI USB-7845R multifunction reconfigurable I/O Device with a frequency of 2.66 kHz. The amplitude and phase of each loudspeaker can be tuned independently. The sound waves emitted by the loudspeakers are guided into the small holes of 6.6 mm in diameter on the bottom of the sample. The positions of the microphone and sample are controlled by the automatic stage. The sound fields are scanned by moving the microphone and sample, with a step size of 7 mm in x direction and 10 mm in y direction. The two ends of the sample are sealed with acoustic foam to avoid unnecessary reflections at boundaries. The information about the geometrical parameters of metasurface and the measuring area is shown in Fig. S6(e).

Considering that the metasurface in Fig. S6(e) could be approximately regarded as the composition of rectangular grooves with the depth $h \approx (26 - R/2)\text{mm} = 24.5\text{mm}$, the width $w = 7\text{mm}$ and the period $a = 11\text{mm}$, the dispersion relation of the acoustic surface wave propagating along the metasurface without losses would be [3–16]:

$$k = \sqrt{\frac{a}{w}\frac{\omega}{c}}\sqrt{1 + \frac{w^2}{a^2}\tan\left(\sqrt{\frac{a}{w}\frac{\omega}{c}}h\right)} \tag{S15}$$

where $c = 343\text{m/s}$ is the acoustic speed in air. The calculated dispersion has been shown in Fig. S6(d), which is similar to spoof surface plasma. The frequency 2660Hz is chosen for the experimental demonstration. It should be mentioned that the closer the working frequency is to the cut-off frequency, the stronger the attenuation would be for surface waves. Because the slow group velocity would correspond to the strong local resonance, which would enhance the intrinsic loss from thermo-viscous damping in narrow channels [17–19].

* These two authors contributed equally to this work.
† xonics@tongji.edu.cn
‡ luminghui@nju.edu.cn

[1] Benalcazar, W. A., Bernevig, B. A. & Hughes, T. L. Quantized electric multipole insulators. *Science* **357**, 61–66 (2017).
[2] Serra-Garcia, M. *et al.* Observation of a phononic quadrupole topological insulator. *Nature* **555**, 342 (2018).
[3] Shi, C. *et al.* Observation of acoustic spin. *Natl. Sci. Rev.* nwz059 (2019).
[4] Zhu, J. *et al.* Acoustic rainbow trapping. *Sci. Rep.* **3**, 1728 (2013).
[5] Lu, J., Qiu, C., Ke, M. & Liu, Z. Directional excitation of the designer surface acoustic waves. *Applied Physics Letters* **106**, 201901 (2015).
[6] Jia, H., Lu, M., Ni, X., Bao, M. & Li, X. Spatial separation of spoof surface acoustic waves on the graded groove grating. *Journal of Applied Physics* **116**, 124504 (2014).
[7] Christensen, J., Fernandez-Dominguez, A., de Leon-Perez, F., Martin-Moreno, L. & Garcia-Vidal, F. Collimation of sound assisted by acoustic surface waves. *Nature Physics* **3**, 851 (2007).
[8] He, Z. *et al.* Nonleaky surface acoustic waves on a textured rigid surface. *Phys. Rev. B* **83**, 132101 (2011).
[9] Kelders, L., Allard, J. F. & Lauriks, W. Ultrasonic surface waves above rectangular-groove gratings. *The Journal of the Acoustical Society of America* **103**, 2730–2733 (1998).
[10] Zhu, J. *et al.* A holey-structured metamaterial for acoustic deep-subwavelength imaging. *Nat. Phys.* **7**, 52 (2011).
[11] Liu, T., Chen, F., Liang, S., Gao, H. & Zhu, J. Subwavelength sound focusing and imaging via gradient metasurface-enabled spoof surface acoustic wave modulation. *Phys. Rev. Appl.* **11**, 034061 (2019).
[12] Zhao, R., Liu, T., Wen, C.-y., Zhu, J. & Cheng, L. Impedance-near-zero acoustic metasurface for hypersonic boundary-layer flow stabilization. *Phys. Rev. Appl.* **11**, 044015 (2019).
[13] Wu, L. T. *et al.* Generation of multiband spoof surface acoustic waves via high-order modes. *Phys. Rev. B* **97**, 214305 (2018).
[14] Liu, T., Zhu, X., Chen, F., Liang, S. & Zhu, J. Unidirectional wave vector manipulation in two-dimensional space with an all passive acoustic parity-time-symmetric metamaterials crystal. *Phys. Rev. Lett.* **120**, 124502 (2018).

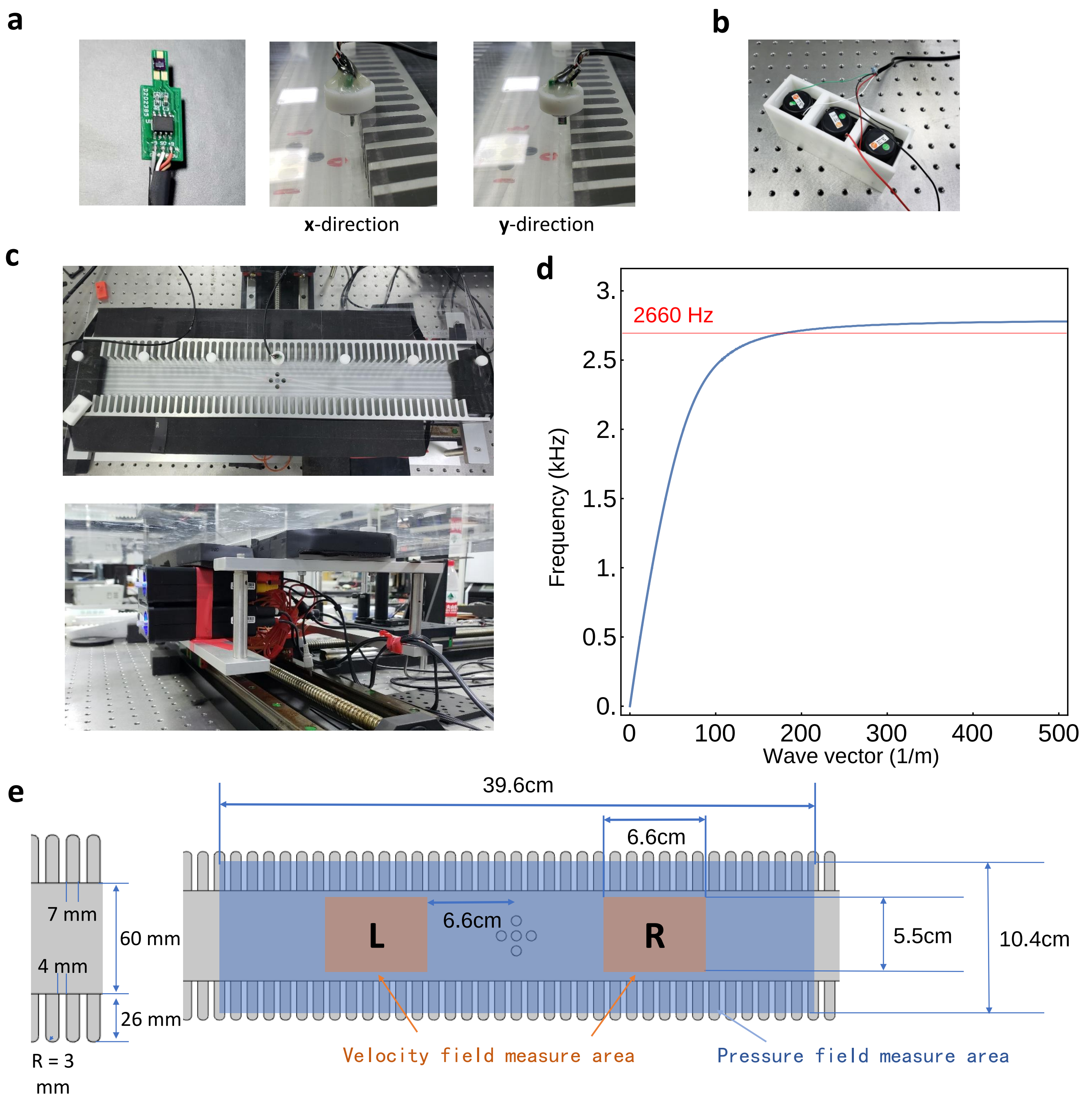

FIG. S6. The experimental setups. (a)The acoustic particle velocity sensor. Different geometrical settings would make the detector measure velocity field with different polarizations. (b) The speakers are controlled by the external circuits. (c) The sound fields are obtained by moving the microphone and sample on the automatic stage. (d) The calculated dispersion of the surface wave on metasurface. (e) The geometrical information about the metasurface structure and measurement area.

[15] Zhou, Y. *et al.* Acoustic surface evanescent wave and its dominant contribution to extraordinary acoustic transmission and collimation of sound. *Phys. Rev. Lett.* **104**, 164301 (2010).

[16] Jia, H., Lu, M., Wang, Q., Bao, M. & Li, X. Subwavelength imaging through spoof surface acoustic waves on a two-dimensional structured rigid surface. *Appl. Phys. Lett.* **103**, 103505 (2013).

[17] Stinson, M. R. The propagation of plane sound waves in narrow and wide circular tubes, and generalization to uniform tubes of arbitrary cross-sectional shape. *The Journal of the Acoustical Society of America* **89**, 550–558 (1991).

[18] Schwan, L., Geslain, A., Romero-García, V. & Groby, J.-P. Complex dispersion relation of surface acoustic waves at a lossy metasurface. *Appl. Phys. Lett.* **110**, 051902 (2017).

[19] Liu, T., Liang, S., Chen, F. & Zhu, J. Inherent losses induced absorptive acoustic rainbow trapping with a gradient metasur-

face. *J Appl. Phys.* **123**, 091702 (2018).
